# Functional characterisation of the osteoarthritis susceptibility locus at chromosome 6q14.1 marked by the polymorphism rs9350591

**DOI:** 10.1186/s12881-015-0215-9

**Published:** 2015-09-07

**Authors:** Katherine Johnson, Louise N. Reynard, John Loughlin

**Affiliations:** Musculoskeletal Research Group, Institute of Cellular Medicine, 4th Floor Catherine Cookson Building, Framlington Place, Newcastle University, Newcastle-upon-Tyne, NE2 4HH UK

## Abstract

**Background:**

The arcOGEN genome-wide association study reported the rs9350591 C/T single nucleotide polymorphism (SNP) as marking a region on chromosome 6q14.1 that is associated with hip osteoarthritis (OA) in Europeans, with an odds ratio (OR) of 1.18 and a *p*-value of 2.42 × 10^−9^. rs9350591 is an intergenic SNP surrounded by seven genes within 1 Mb. Six of the genes are expressed in cartilage. We sought to characterise this signal to assess whether the association of rs9350591 with OA is mediated by modulating gene expression.

**Methods:**

Total RNA was extracted from hip or knee cartilage of 161 OA patients and from hip cartilage of 29 non-OA patients who had undergone hip replacements as a result of neck-of-femur (NOF) fractures. We used quantitative PCR (qPCR) to measure overall gene expression, and pyrosequencing to assess allelic expression of the genes. A mesenchymal stem cell (MSC) differentiation model was used to assess gene expression during chondrogenesis.

**Results:**

We identified a significant decrease in the expression of *SENP6* (*p* = 0.005) and *MYO6* (*p* = 0.026) in OA hip cartilage relative to the non-OA hip control cartilage. However, we found no evidence for a correlation between gene expression and rs9350591 genotype for any of the six genes. In addition, we identified expression quantitative trait loci (eQTLs) operating on *COL12A1*, *TMEM30A*, *SENP6* and *MYO6*, although these were not relevant to the OA associated signal. Finally, all genes were dynamically expressed during chondrogenesis.

**Conclusions:**

The regulation of gene expression at this locus is complex, highlighted by the down-regulation of *SENP6* and *MYO6* in OA hip cartilage and by eQTLs operating on four of the genes at the locus. However, modulation of gene expression in the end-stage OA cartilage that we have investigated is not the mechanism by which this association signal is operating. As implied by the dynamic patterns of gene expression throughout chondrogenesis, the association signal marked by rs9350591 could instead be exerting its effects during joint development.

**Electronic supplementary material:**

The online version of this article (doi:10.1186/s12881-015-0215-9) contains supplementary material, which is available to authorized users.

## Background

Osteoarthritis (OA) is the most common form of arthritis, characterised by the age-related progressive loss of articular cartilage at synovial joints [1]. OA is polygenic, with a large number of small-effect risk-conferring alleles contributing to disease susceptibility [2]. Genome-wide association scans (GWAS) are powerful tools in the identification of susceptibility loci due to their use of large cohorts, independent replication and extensive coverage without bias towards specific regions [3].

In 2012, the arcOGEN GWAS reported five genome-wide significant OA association signals in Europeans [4]. These resulted from the genotyping of almost 1.4 million single nucleotide polymorphisms (SNPs) in a discovery sample of 7,410 cases and 11,009 controls, with replication in 14,883 cases and 53,947 controls. One of the five signals was to the intergenic SNP rs9350591 on chromosome 6q14.1, with an odds ratio (OR) of 1.18 for the minor T allele and a *p*-value of 2.42 × 10^−9^ in the hip stratum. The association signal encompasses six genes that are expressed in cartilage [4]: *COL12A1* coding for the α1 polypeptide chain of type XII collagen, *COX7A2* coding for cytochrome c oxidase 7A2, *TMEM30A* coding for transmembrane protein 30A, *FILIP1* coding for filamin A interacting protein 1, *SENP6* coding for sentrin-specific peptidase 6, and *MYO6* coding for myosin VI.

It is now clear that the majority of common alleles that influence polygenic traits do so by regulating gene expression, so called expression quantitative trait loci, or eQTLs [5, 6]. Most of these act on nearby genes and are termed *cis*-eQTLs. An established example of an OA eQTL is the *GDF5* 5′ untranslated region (UTR) SNP rs143383, the risk-conferring T allele of which correlates with a decrease in *GDF5* expression [7]. More recently, *GNL3* and *SPCS1*, which reside within an OA association signal on chromosome 3p21.1, were shown to be subject to *cis*-acting polymorphisms that influence gene expression in OA joint tissue [8]. Additionally, *DIO2* was subject to the *cis*-eQTL actions of rs225014, the risk allele transcript of which was more abundant in OA cartilage [9].

There are no non-synonymous SNPs residing within the rs9350591 association region that could account for the OA signal as the highest *r*^*2*^ is only 0.085 between rs9350591 and rs17414086 (a C/T SNP in *SENP6* coding for a threonine to methionine substitution). We therefore hypothesised that, like rs143383 and *GDF5*, rs9350591 marks a *cis*-eQTL that accounts for the OA signal at the 6q14.1 locus. To assess this, we investigated the expression of the genes from within the locus in hip and knee cartilage. We first measured overall expression of *COL12A1*, *COX7A2*, *TMEM30A*, *FILIP1*, *SENP6* and *MYO6* using quantitative PCR (qPCR) and stratified the data by disease state and by rs9350591 genotype. We subsequently measured the allelic output of *COL12A1*, *TMEM30A*, *SENP6* and *MYO6* using pyrosequencing to quantify mRNA synthesised from each allelic transcript. We also used an *in vitro* mesenchymal stem cell (MSC) differentiation model to track the expression of the six genes throughout chondrogenesis.

## Methods

### Online database searches

The Broad Institute [10] online software was used to conduct a search of SNPs in high linkage disequilibrium (LD; *r*^*2*^ ≥ 0.8) with the association SNP, rs9350591. The RegulomeDB [11] online database was used to explore the functionality of the polymorphisms.

### Patients

Ethical approval was granted by the Newcastle and North Tyneside research ethics committee (REC reference number 09/H0906/72) to collect tissue from two groups of donors: those with primary OA who had undergone elective total joint replacement of the hip (total hip replacement; THR) or knee (total knee replacement; TKR), and those that had undergone a THR a result of a neck-of-femur (NOF) fracture. The cartilage of the OA patients had visible lesions and these patients were screened to exclude OA due to trauma or other pathologies. The NOF patients showed no signs or symptoms of hip OA, with the cartilage being macroscopically intact and with no lesions. The cartilage was collected from the tibial plateau and the lateral and medial femoral condyles. For the OA patients, the cartilage was collected at sites distal to the OA lesion. Informed written consent for tissue use and data publication was provided by all donors.

### Nucleic acid extraction

Cartilage specimens were snap-frozen at −80 °C on the day of surgery. The cartilage was ground to a powder under liquid nitrogen and genomic DNA and total RNA were extracted using an E.Z.N.A.® DNA/RNA Isolation Kit (Omega Bio-Tek, Georgia, USA), following the manufacturer’s guidelines. Nucleic acid was quantified and cDNA was synthesised as previously described [12].

### Quantitative gene expression analysis

Custom-designed, quality-controlled PrimeTime® TaqMan primers and probes (Integrated DNA Technologies, USA; Additional file 1) were used in real-time qPCR to measure the expression of *COL12A1*, *COX7A2*, *TMEM30A*, *FILIP1*, *SENP6* and *MYO6* in OA and NOF donor cartilage. Patient details can be found in Additional file 2 and *n* numbers can be found in Additional file 3. Reactions were performed in triplicate on an ABI PRISM 7900HT Sequence Detection System (Applied Biosystems, UK), and the data normalised to that of the housekeeping genes *18S*, *GAPDH* and *HPRT1* using the 2^-ΔCt^ method. A Mann–Whitney *U* test was performed to assess if gene expression significantly differed between OA and NOF cohorts, and between rs9350591 genotypes. Given the rarity of T allele homozygotes, gene expression was compared between CC homozygotes and T carriers only.

### SNP selection

Following a search of publicly available databases for known transcript SNPs, we selected two transcript SNPs for allelic expression imbalance analysis per gene for *COL12A1*, *SENP6*, and *MYO6*, and one transcript SNP for *TMEM30A* (Additional file 4). We excluded *COX7A2* as there were no known transcript SNPs, and *FILIP1* because the transcript SNPs had low minor allele frequencies (MAF; < 5 %).

### SNP genotyping

rs9350591 was genotyped by restriction fragment length polymorphism (RFLP) analysis. All transcript SNP genotypes were determined by pyrosequencing. The primer sequences and rs9350591 restriction enzyme used are listed in Additional file 5.

### Allelic expression analysis

We used pyrosequencing to assess allelic expression imbalance (AEI) as previously described [8]. Patient details can be found in Additional file 2 and *n* numbers can be found in Additional file 3. Each pyrosequencing assay was validated using known artificial allelic ratios prior to use in genotyping and AEI analysis. We performed three technical replicates, normalising the cDNA allelic ratios to the mean of the corresponding genomic DNA ratios. A Mann–Whitney *U* test was performed to assess the association between AEI ratios and genotype at rs9350591.

### Mesenchymal stem cells

MSCs from three young human donors were purchased from Lonza, UK. MSCs from three OA patients were harvested from femoral neck aspirates following total hip replacements. Briefly, trabecular bone was extracted and passed through a 100 μm cell strainer in a solution of Dulbecco’s PBS (Life Technologies, UK), 100 U/ml penicillin and 100 μg/ml streptomycin. The cell mixture was layered over 10 ml Ficoll-Paque (GE Healthcare, UK) and centrifuged at 800 *g* for 40 min at room temperature. The buffy coat, containing mononuclear cells, was washed in a solution of Dulbecco’s PBS containing 5 mM EDTA, 0.2 % BSA, 100 U/ml penicillin and 100 μg/ml streptomycin. Cells were pelleted by centrifugation at 200 *g* for 10 min at room temperature. The wash and centrifugation were repeated once again. The cell pellet was resuspended in Dulbecco’s PBS, 100 U/ml penicillin and 100 μg/ml streptomycin. Cells were cultured in a 25 cm^2^ cell culture flask (Sigma-Aldrich, UK) for 24 h before the media, supplemented with 8 ng/ml bFGF (Millipore, UK), was replenished. Additional file 6 provides details regarding the Lonza donors and OA patients from whom MSCs were derived.

### Mesenchymal stem cell differentiation into chondrocytes

Chondrogenesis was induced by culturing the MSCs in a cocktail of media and additives including 10 ng/ml TGF-β3 and 100 nM dexamethasone in a well-established *in vitro* differentiation model [13]. In this model, by day 14 the MSCs have created a cartilaginous disc with an extensive extracellular matrix. RNA was extracted at days 3, 7 and 14 using TRIzol® Reagent (Life Technologies, UK) following the manufacturer’s guidelines and cDNA synthesised as described above.

## Results

### Database searches to investigate the functionality of the region surrounding rs9350591

Although there are no publicly available bioinformatics data pertaining specifically to cartilage, we explored online databases to interrogate the region in related cell lines. We found evidence of functionality, including transcription factor binding and regions with regulatory activity, for SNPs in perfect or high LD with rs9350591 (Additional file 7). All of the 39 SNPs with an *r*^*2*^ of 0.8 or above with rs9350591 are intronic or intergenic, supporting our hypothesis that rs9350591 marks a *cis*-eQTL that accounts for the OA signal at the 6q14.1 locus.

### Quantitative comparison of gene expression in cartilage of hip OA and NOF donors

**Fig. 1:**
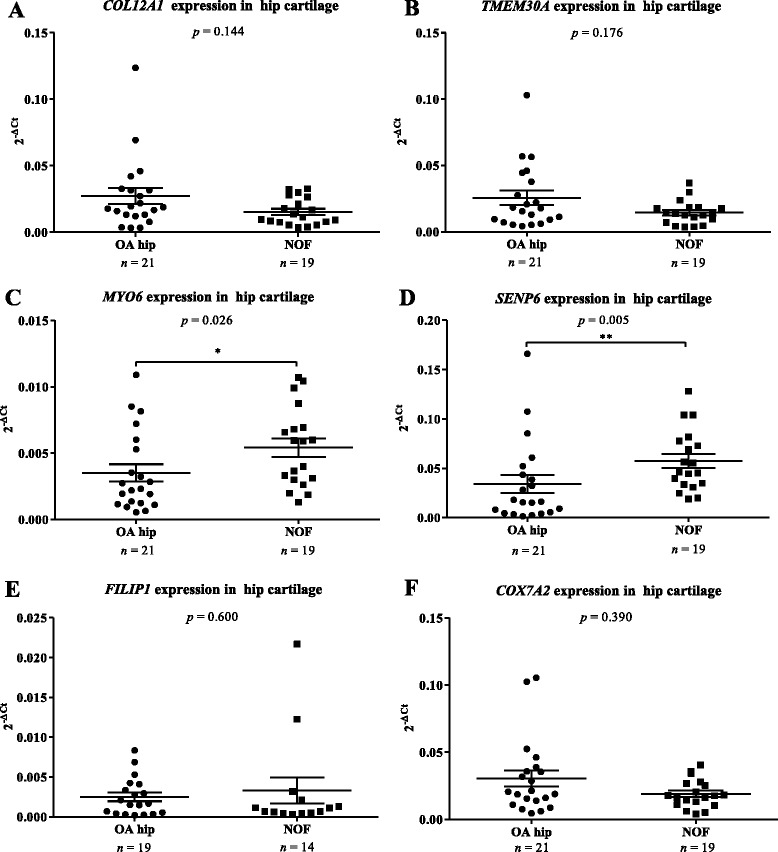
Columnar scatter plots of the expression of the six genes in osteoarthritis (OA) and non-OA hip cartilage. Non-OA hip cartilage was obtained from patients who had undergone a total hip replacement due to a neck-of-femur (NOF) fracture. Gene expression for (**a**) *COL12A1*, (**b**) *TMEM30A*, (**c**) *MYO6*, (**d**) *SENP6*, (**e**) *FILIP1* and (**f**) *COX7A2* was normalised to that of the housekeepers *18S*, *GAPDH* and *HPRT1*. Each dot represents one donor and *n* is the number of donors studied in each group. The horizontal lines represent the mean and the error bars represent the standard error of the mean (SEM). Statistical significance was assessed using the Mann–Whitney *U* test and is not corrected for multiple testing. * *p* < 0.05; ** *p* < 0.01

We quantified *COL12A1*, *COX7A2*, *TMEM30A*, *FILIP1*, *SENP6*, and *MYO6* and compared the expression levels between OA cartilage (knee and hip OA patients combined; *n* ≥ 68) and NOF cartilage (*n* ≥ 14; Additional file 8). This revealed that the expressions of *COL12A1* (*p* = 0.011) and *COX7A2* (*p* = 0.03) were significantly increased and the expression of *SENP6* (*p* = 0.001) significantly decreased in OA. As rs9350591 was identified as being associated with OA in the hip stratum of the arcOGEN study, we next compared gene expression between OA hip and OA knee (*data not shown*). We observed a reduced expression of *MYO6* (*p* = 0.047) in OA hip relative to OA knee. Finally, we removed the OA knee samples from the analyses and directly compared OA hip (*n* ≥ 19) and NOF (*n* ≥ 14; Fig. 1). The expressions of both *SENP6* (*p* = 0.005) and *MYO6* (*p* = 0.026) were reduced in OA hip relative to NOF.

### Quantitative comparison of gene expression in cartilage stratified by rs9350591 genotype

As highlighted above, all of the SNPs that can account for the association signal—that is, those that are in an LD of ≥ 0.8 with rs9350591—are intronic or intergenic and we can therefore discount the action of a non-synonymous SNP altering protein structure or function. This implies that rs9350591 marks a functional role for one of the 39 polymorphisms in the form of a *cis*-eQTL. The fact that the SNPs are in perfect or high LD with the association signal (all have a *D’* of 1 with rs9350591; Additional file 7) negates the need to genotype all of the polymorphisms.

To assess if the OA association was acting as an eQTL on one or more of the six genes, we therefore quantified overall levels of gene expression in OA cartilage (knee and hip OA patients combined) and stratified the data by rs9350591 genotype. Of the 74 OA donors whom we assayed, only two were homozygous for the minor T allele of rs9350951. This is in close agreement with the calculated HapMap CEU rs9350591 TT genotype frequency of 1.7 %. For a robust analysis, we therefore grouped all T allele carriers (*n* ≥ 23), that is, minor allele homozygotes (TT) and heterozygotes (CT), and compared these to major allele homozygotes (CC; *n* ≥ 45). We observed no significant differences in cartilage gene expression that correlated with rs9350591 genotype (Additional file 9).

**Fig. 2:**
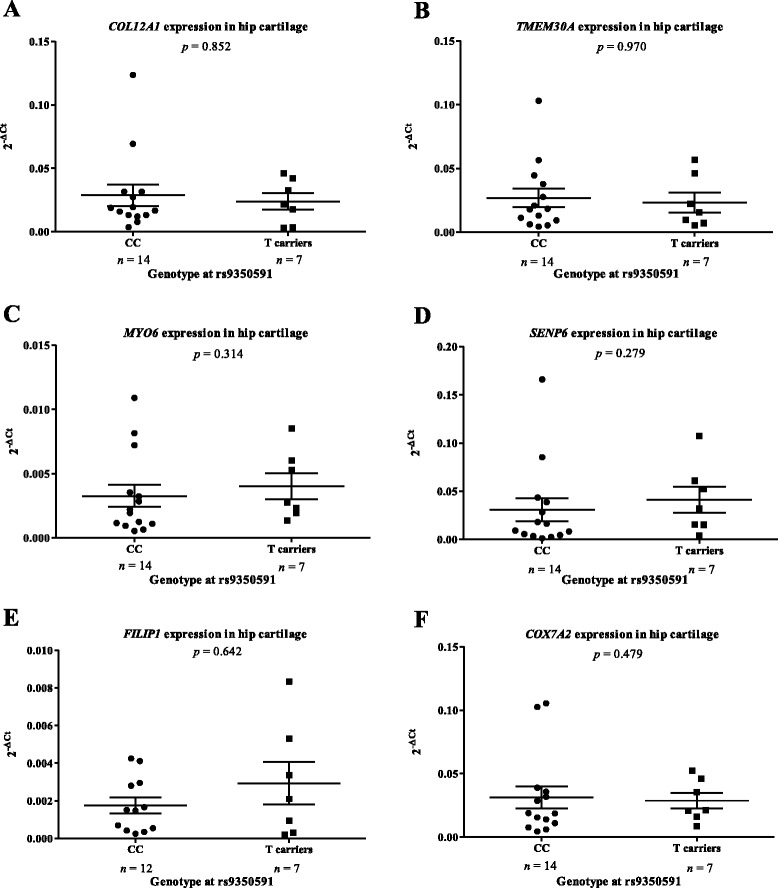
Columnar scatter plots of the expression of the six genes in osteoarthritis (OA) hip cartilage stratified by genotype at the association SNP rs9350591. Gene expression for (**a**) *COL12A1*, (**b**) *TMEM30A*, (**c**) *MYO6*, (**d**) *SENP6*, (**e**) *FILIP1* and (**f**) *COX7A2* was normalised to that of the housekeepers *18S*, *GAPDH* and *HPRT1*. Each dot represents one donor and *n* is the number of donors studied in each group. CC is the major allele homozygote genotype at the association SNP rs9350591, whilst T carriers are those that are either heterozygote (CT) or minor allele homozygote (TT) at the SNP. The horizontal lines represent the mean and the error bars represent the standard error of the mean (SEM). Statistical significance was assessed using the Mann–Whitney *U* test and is not corrected for multiple testing

**Fig. 3:**
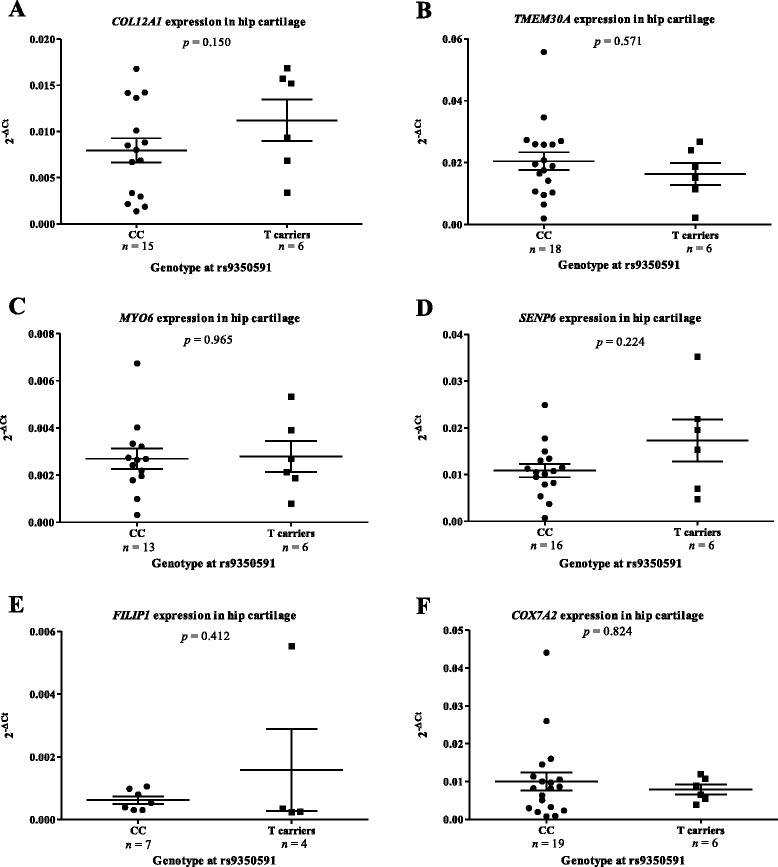
Columnar scatter plots of the expression of the six genes in a replication cohort of osteoarthritis (OA) hip cartilage stratified by genotype at the association SNP rs9350591. Gene expression for (**a**) *COL12A1*, (**b**) *TMEM30A*, (**c**) *MYO6*, (**d**) *SENP6*, (**e**) *FILIP1* and (**f**) *COX7A2* was normalised to that of the housekeepers *18S*, *GAPDH* and *HPRT1*. Each dot represents one donor and *n* is the number of donors studied in each group. CC is the major allele homozygote genotype at the association SNP rs9350591, whilst T carriers are those that are either heterozygote (CT) or minor allele homozygote (TT) at the SNP. The horizontal lines represent the mean and the error bars represent the standard error of the mean (SEM). Statistical significance was assessed using the Mann–Whitney *U* test and is not corrected for multiple testing. * *p* < 0.05

Again, we removed the OA knee data to allow for the comparison of OA hip donors only (CC *n* ≥ 12; T carriers *n* = 7; Fig. 2). We observed no significant differences that correlated with rs9350591 genotype. To corroborate these findings, we replicated the analysis in an independent cohort of 25 additional OA hip donors (CC *n* ≥ 7; T carriers *n* ≥ 4; Fig. 3), and again observed no significant correlations of expression with rs9350591 genotype for any gene.

### Allelic expression imbalance analysis of *COL12A1*, *TMEM30A*, *SENP6* and *MYO6* in cartilage

**Fig. 4:**
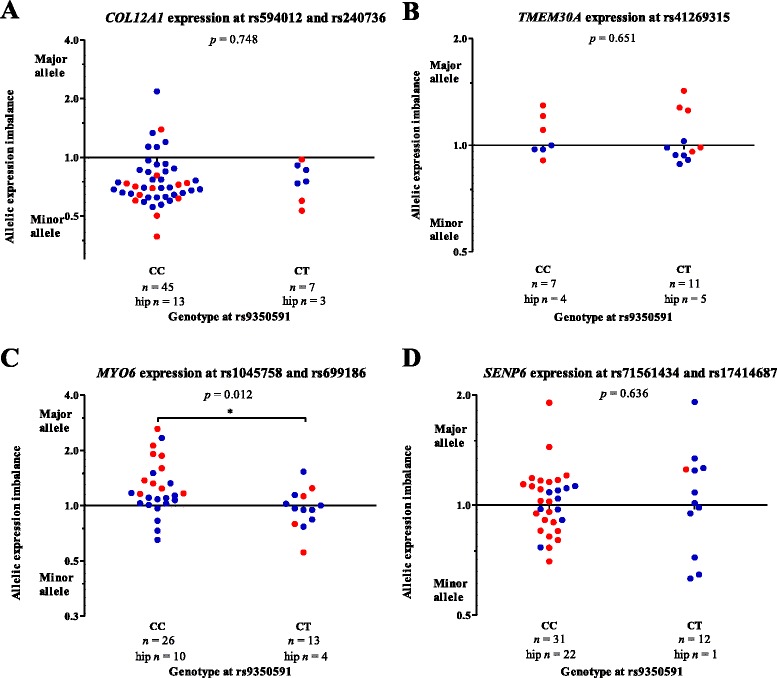
Columnar scatter plots of allelic expression imbalance (AEI) for four genes in osteoarthritis (OA) cartilage stratified by genotype at the association SNP rs9350591. AEI for (**a**) *COL12A1*, (**b**) *TMEM30A*, (**c**) *MYO6* and (**d**) *SENP6*. Each dot represents one donor and *n* is the number of donors studied in each group. CC is the major allele homozygote genotype at rs9350591 whilst CT is the heterozygote genotype at this SNP. Red dots are hip (OA and NOF) cartilage and blue dots are OA knee cartilage. Patients showing allelic expression balance have a value of one on the *y*-axis whilst deviations from this indicate allelic expression imbalance. Statistical significance was assessed using the Mann–Whitney *U* test and is not corrected for multiple testing. * *p* < 0.05

Correlating genotype with overall gene expression is vulnerable to the natural fluctuation in gene expression, such that subtle genotypic effects on gene expression may be hidden by inter-individual variation in gene expression, generating false-negative results. Directly testing for allelic expression differences within an individual can overcome this. We therefore identified patients who were heterozygous for transcript SNPs, measured allelic expression by pyrosequencing in cartilage from these individuals and then stratified this data by genotype at rs9350591. If this association SNP correlates with gene expression, then AEI would be observed in compound heterozygotes, i.e. individuals heterozygous for both the transcript SNP and rs9350591, and the AEI ratios would be significantly different to any AEI ratios observed in individuals homozygous for rs9350591 (Additional file 10). We were able to design and validate AEI assays for transcript SNPs within *COL12A1* (rs594012 and rs240736), *TMEM30A* (rs41269315), *SENP6* (rs71561434 and rs17414687) and *MYO6* (rs1045758 and rs699186). We were unable to perform AEI analysis for *COX7A2*, which has no known transcript SNPs, or for *FILIP1*, whose transcript SNPs have MAFs < 5 %, which precluded the identification of a sufficiently large number of heterozygotes to do a meaningful analysis upon. As we aimed to investigate the effect of rs9350591 genotype specifically on allelic output, it was not necessary to stratify the data by disease state or joint site; however to ensure a thorough investigation, we have included this analysis in Additional file 11. None of the patients used in this AEI analysis were TT homozygotes at rs9350591 and our analysis of each of the four genes therefore involved a comparison of patients who were all heterozygous at a transcript SNP for that gene and either homozygous CC (*n* ≥ 7) or heterozygous CT (*n* ≥ 7) at rs9350591. We identified a spread of allelic expression ratios for all four genes (Fig. 4), which therefore supported the activity of eQTLs operating at this locus. The AEI was greatest for *MYO6*, with allelic ratios in excess of 1.5 for some individuals. However, these eQTL activities were observed in cartilage from individuals homozygous for rs9350591 as well as heterozygotes, and as such are not dependent on genotype at the association SNP. There were no significant differences (*p* < 0.05) in the AEI ratios between CC and CT patients for *COL12A1*, *TMEM30A* or *SENP6*. There was a significant difference (*p* = 0.019; Additional file 11) for *MYO6* AEI in OA hip cartilage, which remained significant upon combining the data with OA knee and NOF (*p* = 0.012; Fig. 4), although this was accounted for by a large allelic ratio spread in those patients homozygous CC for rs9350591. Therefore, the AEI of *MYO6* is not correlating with the eQTL marked by rs9350591. Combined with the large number of statistical tests that we have performed, a *p* of 0.012 would not endure if corrected for multiple testing; this implies that rs9350591 does not correlate with AEI at any of the tested genes.

### Quantitative comparison of gene expression during chondrogenesis

**Fig. 5:**
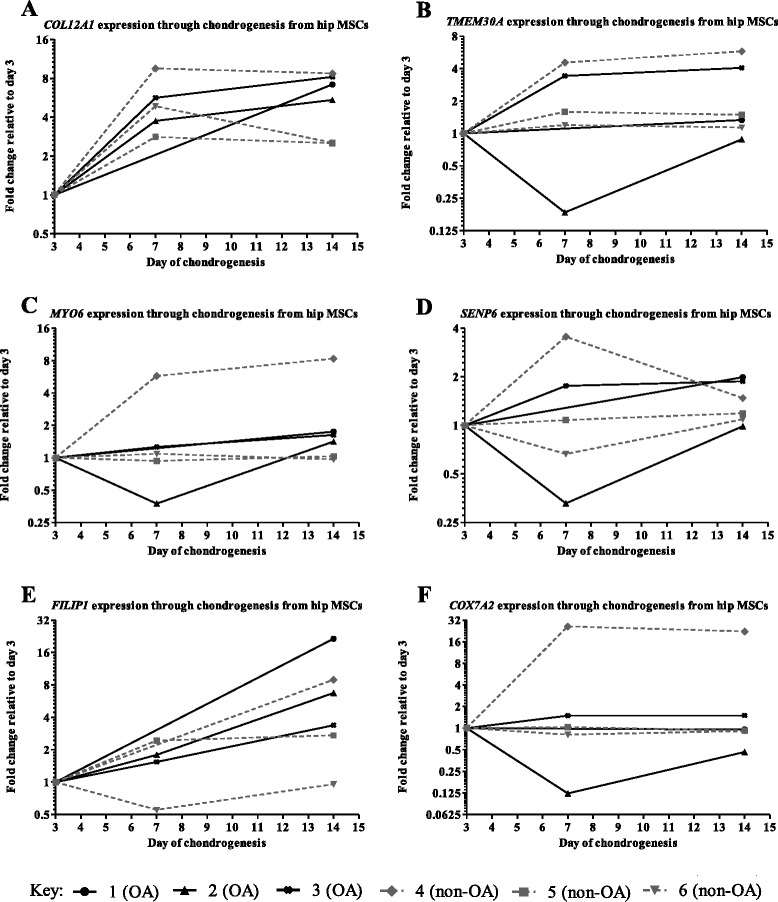
Gene expression throughout chondrogenesis in an *in vitro* mesenchymal stem cell (MSC) differentiation model. Gene expression for (**a**) *COL12A1*, (**b**) *TMEM30A*, (**c**) *MYO6*, (**d**) *SENP6*, (**e**) *FILIP1* and (**f**) *COX7A2* was normalised to that of the housekeepers *18S*, *GAPDH* and *HPRT1*. Measures were taken from discs harvested at days 3, 7 and 14

Our data thus far suggests that the OA association marked by rs9350591 does not correlate with eQTLs operating in the tested genes in the patient cartilage tissues analysed. Although OA is a disease in which age is a predominant risk factor [1], it has been suggested that several OA genetic susceptibility loci may act either during skeletal development or throughout life [14]. The OA association marked by rs9350591 could therefore be exerting its effect on one or more of the nearby genes at an earlier stage of cartilage development. Thus, to assess their potential activity during development, we tracked the expression of the six genes throughout chondrogenesis in a well-established *in vitro* chondrogenesis model. We studied MSCs from three OA and three non-OA (Lonza) donors (Additional file 6). All six genes were expressed throughout chondrogenesis for both types of donor (Fig. 5). Although there were inter-individual variations in the levels of expression, there were no consistent differences between OA and non-OA. The six genes are therefore active during cartilage formation.

## Discussion

The action of *cis*-eQTLs has been shown to be an important mechanism by which OA susceptibility is mediated. In addition to the *GDF5*, *GNL3/SPCS1* and *DIO2* examples given in the Introduction [7–9], one other example is the correlation between reduced *HBP1* expression in cartilage and the presence of the OA association alleles of rs3815148 and rs4730250 at the chromosome 7q22 locus [15]. On this basis, we assessed whether the OA association signal marked by rs9350591 similarly acted as a *cis*-eQTL by modulating the expression of one or more of the six cartilage-expressed genes residing at the locus.

Our initial analysis revealed a significant increase in *COL12A1* and *COX7A2* expression and a decrease in *SENP6* expression in combined OA hip and OA knee cartilage relative to the non-OA NOF (hip) cartilage. When the analysis was repeated for OA hip cartilage only, we observed a significant decrease in *MYO6* and *SENP6* expression compared to NOF. From the perspective of cartilage and OA, none of these four genes have reported roles in skeletal development or joint biology, and so we are unable to identify a single OA candidate gene for further functional analyses. For example, *MYO6* encodes the myosin VI protein, an actin-based motor protein involved in vesicle trafficking [16] and with pathologies related to autosomal dominant hearing loss [17]. However, a role for the protein encoded by *SENP6* could be more relevant. Small ubiquitin-like modifier (SUMO) proteins can covalently attach to other cellular proteins in order to regulate their function in a reversible process known as sumoylation. SENP6 has been shown to control SUMO regulation by deconjugating the proteins from their targets [18], and the emerging evidence of a role of sumoylation in arthritis [19] may make it reasonable to hypothesise that a decrease in *SENP6* expression could lead to altered downstream protein regulation and thus be implicated in OA aetiology.

Our analysis of overall gene expression stratified by genotype did not support a correlation between expression and the association signal for any of the six genes tested. This was also the case when we directly assessed allelic expression in the four genes where this was possible. These four genes did however have eQTLs operating on their expression, but these were unrelated to the association signal. We have to conclude therefore that in the cartilage tissue that we have investigated, from the elderly patients who form our study group, variation in expression of the genes is not an OA risk factor.

All six genes demonstrated dynamic expression patterns during chondrogenesis using both OA and younger MSCs. We speculate therefore that they and their encoded proteins have functional roles during cartilage formation. Based on this, it is reasonable to hypothesise that the association signal could be operating as an eQTL on one or more of these genes during skeletogenesis. Directly testing this hypothesis will however be arduous due to the scarcity of relevant patient tissues. Alternatively, the association signal may be operating on one or more of the six genes but in non-cartilaginous tissues, or on genes outside of the association interval. Further analyses are therefore merited but with a focus away from end-stage diseased cartilage.

## Conclusion

In summary, our data reveal a down-regulation of *SENP6* and *MYO6* in OA hip cartilage relative to non-OA controls. Investigations should be undertaken to further understand, in particular, how SENP6 functions in OA aetiology. We have identified *COL12A1*, *TMEM30A*, *SENP6* and *MYO6* as being subject to eQTLs in cartilage, however, these act independently of rs9350591. In addition, we have demonstrated the diverse patterns of gene expression during cartilage development. These findings suggest that the OA susceptibility of this locus is likely established in an earlier stage of joint development. Overall, our results justify further interrogation of this region.

### Availability of data and materials

The raw data supporting the findings presented here are available from the corresponding author upon request. They are not stored in a publicly available repository.

## Additional files

Additional file 1:
**Primer and probe sequences used for real-time reverse transcription PCR (qPCR).** The PrimeTime® qPCR Assays were purchased from Integrated DNA Technologies (IDT), who perform quality control by mass spectrometry, with all QC results provided on their website. Additional file 2:
**Table of patient characteristics and their genotype at rs9350591 used in gene expression and allelic expression analysis.** OA (donors 1–161) and NOF (donors 162–190). (PDF 158 kb) Additional file 3:
***n*** **numbers used for real-time reverse transcription PCR (qPCR) and allelic expression imbalance (AEI).**
Additional file 4:
**The seven transcript SNPs and their pair-wise** ***D’*** **and** ***r*** ^***2***^ **values relative to rs9350591.**
Additional file 5:
**Primer sequences used for genotyping and allelic expression imbalance by pyrosequencing (top table), and primer sequences and restriction enzyme used for genotyping by restriction fragment length polymorphism analysis (bottom table).**
Additional file 6:
**Table of OA and non-OA (Lonza) MSC donor characteristics used in chondrogenesis.**
Additional file 7:
**Bioinformatics database search of the SNPs in high LD with rs9350591.**
Additional file 8:
**OA vs NOF analysis of qPCR data for hip and knee cartilage combined.** (PDF 75 kb) Additional file 9:
**Genotype analysis of qPCR data for hip and knee cartilage combined.** (PDF 64 kb) Additional file 10:
**Diagram to explain the premise of allelic expression imbalance analysis when there are no transcript SNPs in high LD** **(** ***r*** ^***2***^ **≥ 0.8) with the association SNP.** (PDF 89 kb) Additional file 11:
**Allelic expression imbalance analysis stratified by disease state and joint site.** (PDF 57 kb)

## Abbreviations

AEI: Allelic expression imbalance
eQTL: Expression quantitative trait locus
GWAS: Genome-wide association scan
LD: Linkage disequilibrium
MAF: Minor allele frequency
MSC: Mesenchymal stem cell
NOF: Neck-of-femur
OA: Osteoarthritis
OR: Odds ratio
qPCR: Quantitative polymerase chain reaction
RFLP: Restriction fragment length polymorphism
SEM: Standard error of the mean
SNP: Single nucleotide polymorphism
THR: Total hip replacement
TKR: Total knee replacement
UTR: Untranslated region
SUMO: Small ubiquitin-like modifier
