# Supplementary material for: Functional characterisation of the osteoarthritis susceptibility locus at chromosome 6q14.1 marked by the polymorphism rs9350591
Source: BMC Med Genet. 2015 Sep 7;16:81. doi: 10.1186/s12881-015-0215-9 (PMC4562116; doi:10.1186/s12881-015-0215-9)
Supplement: Additional file 1: — Primer and probe sequences used for real-time reverse transcription PCR (qPCR). The PrimeTime® qPCR Assays were purchased from Integrated DNA Technologies (IDT), who perform quality control by mass spectrometry, with all QC results provided on their website. [file 12881_2015_215_MOESM1_ESM.pdf]

Additional file 1. Primer and probe sequences used for real-time reverse transcription PCR (qPCR). The PrimeTime® qPCR Assays were purchased from Integrated DNA Technologies (IDT), who perform quality control by mass spectrometry, with all QC results provided on their website.

**Gene**	**Probe sequence (5′-3′)**	**Primer 1 (5′-3′)**	**Primer 2 (5′-3′)**
COL12A1	56-FAM/TCCCCTGTG/ZEN/GAAGGCTGATAAGTGA/3IABkFQ	CGGTGATAGTGTAAGGAGTGTC	TGGTCGTGTGCAGAAATATAGG
TMEM30A	56-FAM/TGTGACATT/ZEN/CAAAGAGTATCGGCCAGC/3IABkFQ	CAGCATTACCTACTTTTCGCAAG	TCATCCGTTTTCGTCCATCAA
MYO6	56-FAM/TGGCGTCCT/ZEN/GCACCTTGGAAATA/3IABkFQ	CCAAACCCAGTAATTCAGCAC	AAAGCTTGATCTCTTCCGGG
SENP6	56-FAM/CTGTAAGGT/ZEN/TAAGTCGGCTCCAAGGT/3IABkFQ	TCCTCTTAATTTCAGGCTCCAC	AAAGAATACCCACCTCATGTCC
FILIP1	56-FAM/ACAACGTCA/ZEN/TCTGCTCGAGGAACC/3IABkFQ	AGCACTATCACCATAACACCG	CTTTTGACATAGGAATGCGGG
COX7A2	56-FAM/AGATTGGGC/ZEN/AGAGGACGATAAGCAC/3IABkFQ	TGGTCAGTAACAGCCAAGATG	TTTTAAAATGCCTGCGGGAAG
GAPDH	56-FAM/AAGGTCGGAGTCAACGGATTTGGTC/IABkFQ/36-TAMSp	TGTAGTTGAGGTCAATGAAGGG	ACATCGCTCAGACACCATG
HPRT1	56-FAM/AGGACTGAACGTCTTGCTCGAGATG/36-TAMSp	ACAGAGGGCTACAATGTGATG	TGCTGAGGATTTGGAAAGGG
18S	56-FAM/TCCTTTGGTCGCTCGCTCCTCTCCC/TAMRA	TATTAGCTCTAGAATTACCACAGTTATCC	CGAATGGCTCATTAAATCAGTTATGG
