# Supplementary material for: Functional characterisation of the osteoarthritis susceptibility locus at chromosome 6q14.1 marked by the polymorphism rs9350591
Source: BMC Med Genet. 2015 Sep 7;16:81. doi: 10.1186/s12881-015-0215-9 (PMC4562116; doi:10.1186/s12881-015-0215-9)
Supplement: Additional file 2: — Table of patient characteristics and their genotype at rs9350591 used in gene expression and allelic expression analysis. OA (donors 1–161) and NOF (donors 162–190). (PDF 158 kb) [file 12881_2015_215_MOESM2_ESM.pdf]

Additional file 2. Table of patient characteristics and their genotype at rs9350591 used in gene expression and allelic expression analysis. OA (donors 1-161) and NOF (donors 162-190)

Patient	Sex	Age at surgery (years)	Joint replaced at surgery	Genotype at rs9350591	Used in discovery qPCR?						Used in replication qPCR?						Used in allelic expression imbalance?			
					COL12A1	*TMEM30A*	*MYO6*	*SENP6*	*FILIP1*	*COX7A2*	*COL12A1*	*TMEM30A*	*MYO6*	*SENP6*	*FILIP1*	*COX7A2*	*COL12A1*	*TMEM30A*	*MYO6*	*SENP6*
1	F	75	H	CC	Yes	Yes	Yes	Yes	Yes	Yes										
2	F	67	K	CT														Yes	Yes	Yes
3	M	69	K	CC	Yes	Yes	Yes	Yes	Yes	Yes										
4	F	77	H	CT	Yes	Yes	Yes	Yes	Yes	Yes										
5	F	73	K	CC	Yes	Yes	Yes	Yes	Yes	Yes										
6	M	71	K	CC	Yes	Yes	Yes	Yes		Yes										
7	F	73	K	CC	Yes	Yes	Yes	Yes	Yes	Yes										
8	F	51	H	CT	Yes	Yes	Yes	Yes	Yes	Yes										
9	M	71	H	CC	Yes	Yes	Yes	Yes	Yes	Yes										
10	F	60	K	CC	Yes	Yes	Yes	Yes	Yes	Yes										
11	F	67	K	CT	Yes	Yes	Yes	Yes	Yes	Yes										
12	M	74	K	CC	Yes	Yes	Yes	Yes	Yes	Yes										
13	F	50	K	CC	Yes	Yes	Yes	Yes	Yes	Yes										
14	F	76	H	TT	Yes	Yes	Yes	Yes	Yes	Yes										
15	F	71	K	CT	Yes	Yes	Yes	Yes	Yes	Yes										
16	F	67	H	CC	Yes	Yes	Yes	Yes	Yes	Yes										
17	F	70	H	CC	Yes	Yes	Yes	Yes	Yes	Yes										
18	F	70	H	CC	Yes	Yes	Yes	Yes	Yes	Yes										
19	F	60	H	CC	Yes	Yes	Yes	Yes		Yes										
20	F	78	H	CT	Yes	Yes	Yes	Yes	Yes	Yes										
21	F	81	K	CT	Yes	Yes	Yes	Yes	Yes	Yes										
22	M	76	K	CC	Yes	Yes	Yes	Yes	Yes	Yes							Yes		Yes	Yes
23	F	55	K	CT	Yes	Yes	Yes	Yes	Yes	Yes									Yes	
24	M	57	K	CC	Yes	Yes	Yes	Yes	Yes	Yes									Yes	
25	M	57	H	CC	Yes	Yes	Yes	Yes	Yes	Yes										
26	M	69	K	CC	Yes	Yes	Yes	Yes	Yes	Yes										
27	M	74	K	CC	Yes	Yes	Yes	Yes	Yes	Yes										
28	F	67	K	CT	Yes	Yes	Yes	Yes	Yes	Yes								Yes	Yes	Yes
29	F	69	K	CC	Yes	Yes	Yes	Yes	Yes	Yes										
30	M	57	K	CT	Yes	Yes	Yes	Yes	Yes	Yes										
31	F	61	H	CC	Yes	Yes	Yes	Yes	Yes	Yes										
32	F	66	K	CT	Yes	Yes	Yes	Yes	Yes	Yes										
33	M	63	K	CC	Yes	Yes	Yes	Yes	Yes	Yes										
34	M	77	K	CT													Yes			
35	M	82	K	CT	Yes	Yes	Yes	Yes	Yes	Yes										
36	F	78	K	CC	Yes	Yes	Yes	Yes	Yes	Yes										
37	M	82	K	CC	Yes	Yes	Yes	Yes	Yes	Yes										
38	M	56	K	CC	Yes	Yes	Yes	Yes	Yes	Yes										
39	F	54	K	CC	Yes	Yes	Yes	Yes	Yes	Yes							Yes			
40	M	71	K	CC	Yes	Yes	Yes	Yes	Yes	Yes										
41	F	58	H	CC	Yes	Yes	Yes	Yes	Yes	Yes										
42	F	69	H	CC	Yes	Yes	Yes	Yes	Yes	Yes										
43	M	63	K	CT	Yes	Yes	Yes	Yes	Yes	Yes								Yes		
44	F	71	H	CT	Yes	Yes	Yes	Yes	Yes	Yes	Yes	Yes	Yes	Yes	Yes	Yes				
45	M	70	K	CC	Yes	Yes	Yes	Yes	Yes	Yes										
46	M	67	K	CC	Yes	Yes	Yes	Yes	Yes	Yes										
47	M	86	K	CC	Yes	Yes	Yes	Yes	Yes	Yes							Yes		Yes	
48	F	80	K	TT	Yes	Yes	Yes	Yes	Yes	Yes										

Patient	Sex	Age at surgery (years)	Joint replaced at surgery	Genotype at rs9350591	Used in discovery qPCR?						Used in replication qPCR?						Used in allelic expression imbalance?			
					COL12A1	*TMEM30A*	*MYO6*	*SENP6*	*FILIP1*	*COX7A2*	*COL12A1*	*TMEM30A*	*MYO6*	*SENP6*	*FILIP1*	*COX7A2*	*COL12A1*	*TMEM30A*	*MYO6*	*SENP6*
49	M	71	K	CT	Yes	Yes	Yes	Yes	Yes	Yes										
50	F	67	K	CC													Yes			
51	F	58	K	CC	Yes	Yes	Yes	Yes	Yes	Yes										
52	M	59	K	CT	Yes	Yes	Yes	Yes	Yes	Yes										
53	F	81	K	CT	Yes	Yes	Yes	Yes	Yes	Yes							Yes		Yes	Yes
54	F	80	K	CC	Yes	Yes	Yes	Yes		Yes									Yes	
55	F	64	K	CC													Yes			
56	F	62	K	CC	Yes	Yes	Yes	Yes	Yes	Yes										
57	F	80	K	CT	Yes	Yes	Yes	Yes	Yes	Yes								Yes		
58	F	59	K	CC	Yes	Yes	Yes	Yes	Yes	Yes							Yes			
59	F	71	H	CT							Yes	Yes	Yes	Yes	Yes	Yes				
60	M	74	K	CC	Yes	Yes	Yes	Yes	Yes	Yes										
61	M	72	K	CC	Yes	Yes	Yes	Yes	Yes	Yes										
62	M	72	K	CC	Yes	Yes	Yes	Yes	Yes	Yes										
63	M	68	H	CC	Yes	Yes	Yes	Yes	Yes	Yes										
64	F	63	H	CC	Yes	Yes	Yes	Yes	Yes	Yes										
65	M	77	K	CC	Yes	Yes	Yes	Yes	Yes	Yes										
66	M	71	H	CC	Yes	Yes	Yes	Yes	Yes	Yes										
67	M	71	H	CT	Yes	Yes	Yes	Yes	Yes	Yes										
68	F	73	K	CC	Yes	Yes	Yes	Yes	Yes	Yes										
69	F	82	K	CT	Yes	Yes	Yes	Yes	Yes	Yes										
70	M	71	K	CC	Yes	Yes	Yes	Yes	Yes	Yes										
71	M	80	K	CC	Yes	Yes	Yes	Yes	Yes	Yes									Yes	
72	M	66	H	CC	Yes	Yes	Yes	Yes	Yes	Yes	Yes					Yes				
73	M	69	K	CC	Yes	Yes	Yes	Yes		Yes										
74	F	58	K	CC	Yes	Yes	Yes	Yes		Yes										
75	F	60	K	CC	Yes	Yes	Yes	Yes	Yes	Yes										
76	F	62	K	CC	Yes	Yes	Yes	Yes	Yes	Yes										
77	F	67	H	CT	Yes	Yes	Yes	Yes	Yes	Yes										
78	M	53	K	CC													Yes			
79	M	65	K	CC															Yes	
80	M	85	H	CC													Yes		Yes	Yes
81	F	71	K	CC														Yes		
82	M	49	K	CT																Yes
83	F	62	H	CC													Yes		Yes	
84	M	67	K	CC															Yes	
85	F	64	K	CC															Yes	Yes
86	M	59	H	CC								Yes		Yes						
87	M	65	K	CC														Yes		
88	F	72	K	CT																Yes
89	F	54	K	CT																Yes
90	F	72	K	CT													Yes		Yes	Yes
91	F	76	H	CC													Yes		Yes	Yes
92	F	71	K	CC													Yes			Yes
93	M	48	H	CT															Yes	Yes
94	F	59	H	CC							Yes	Yes	Yes	Yes	Yes	Yes	Yes	Yes	Yes	Yes
95	M	90	H	CC							Yes	Yes	Yes	Yes	Yes	Yes				
96	F	52	H	CT															Yes	
97	F	73	K	CC															Yes	
98	F	64	K	CC													Yes		Yes	Yes

Patient	Sex	Age at surgery (years)	Joint replaced at surgery	Genotype at rs9350591	Used in discovery qPCR?						Used in replication qPCR?						Used in allelic expression imbalance?			
					COL12A1	*TMEM30A*	*MYO6*	*SENP6*	*FILIP1*	*COX7A2*	*COL12A1*	*TMEM30A*	*MYO6*	*SENP6*	*FILIP1*	*COX7A2*	*COL12A1*	*TMEM30A*	*MYO6*	*SENP6*
99	M	69	K	CC																Yes
100	F	68	K	CT														Yes	Yes	Yes
101	M	78	H	CT							Yes	Yes	Yes	Yes	Yes	Yes		Yes		Yes
102	F	88	K	CT															Yes	Yes
103	F	61	K	CC																Yes
104	F	70	H	CT													Yes			
105	F	70	H	CT							Yes	Yes	Yes	Yes		Yes				
106	F	76	K	CC													Yes		Yes	
107	M	58	H	CC																Yes
108	M	54	K	CT															Yes	
109	M	54	K	CT															Yes	
110	F	66	K	CC													Yes			
111	F	78	K	CC															Yes	Yes
112	M	73	K	CC													Yes			
113	F	67	H	CC										Yes		Yes	Yes			
114	M	58	K	CC													Yes		Yes	
115	F	45	H	CC								Yes				Yes	Yes			Yes
116	F	67	H	CC													Yes		Yes	
117	F	45	H	CC																Yes
118	F	68	K	CC													Yes		Yes	
119	M	82	K	CC													Yes		Yes	
120	F	60	K	CT																Yes
121	M	79	K	CT														Yes		Yes
122	M	67	K	CC															Yes	Yes
123	F	68	H	CC							Yes	Yes	Yes	Yes	Yes	Yes	Yes			
124	F	63	K	CC													Yes			
125	F	87	K	CC																Yes
126	F	57	K	CC													Yes			
127	M	68	K	CC													Yes			
128	M	75	K	CC													Yes			
129	F	57	K	CC													Yes			
130	M	55	K	CC													Yes			
131	M	62	K	CC													Yes			
132	F	61	K	CT														Yes		
133	F	54	K	CC													Yes			
134	M	55	K	CC													Yes			
135	M	50	K	CC													Yes			
136	F	56	K	CC													Yes			
137	F	73	K	CC													Yes			
138	F	73	K	CC													Yes			
139	F	56	K	CT													Yes			
140	F	66	H	CC							Yes	Yes	Yes	Yes	Yes	Yes			Yes	Yes
141	F	66	H	CC																
142	F	76	K	CC													Yes			
143	F	89	H	CC							Yes	Yes	Yes	Yes	Yes	Yes				
144	F	64	K	CC													Yes			
145	F	59	H	CC							Yes	Yes				Yes				Yes
146	M	74	H	CT							Yes	Yes	Yes	Yes		Yes	Yes		Yes	
147	F	76	H	CC							Yes	Yes	Yes	Yes		Yes	Yes			
148	F	68	K	CC													Yes			

Patient	Sex	Age at surgery (years)	Joint replaced at surgery	Genotype at rs9350591	Used in discovery qPCR?						Used in replication qPCR?						Used in allelic expression imbalance?			
					COL12A1	*TMEM30A*	*MYO6*	*SENP6*	*FILIP1*	*COX7A2*	*COL12A1*	*TMEM30A*	*MYO6*	*SENP6*	*FILIP1*	*COX7A2*	*COL12A1*	*TMEM30A*	*MYO6*	*SENP6*
149	77	F	H	CC							Yes	Yes	Yes	Yes	Yes	Yes				
150	62	F	H	CC							Yes	Yes	Yes	Yes		Yes				
151	79	F	H	CT							Yes	Yes	Yes	Yes	Yes	Yes				
152	73	M	H	CC							Yes	Yes	Yes	Yes		Yes				
153	51	F	H	CC													Yes			
154	51	F	H	CC							Yes	Yes	Yes	Yes	Yes	Yes				
155	64	M	H	CC								Yes				Yes				
156	58	F	H	CC							Yes	Yes	Yes	Yes		Yes				
157	58	F	H	CC							Yes	Yes	Yes	Yes		Yes				
158	80	F	H	CC							Yes	Yes	Yes	Yes		Yes				
159	84	M	H	CC								Yes		Yes		Yes				
160	M	68	K	CT	Yes	Yes	Yes	Yes	Yes	Yes										
161	M	75	K	CT	Yes	Yes	Yes	Yes		Yes										
162	F	69	H	CT	Yes	Yes	Yes	Yes	Yes	Yes										
163	F	71	H	CC	Yes	Yes	Yes	Yes	Yes	Yes										
164	F	81	H	CC	Yes	Yes	Yes	Yes	Yes	Yes										
165	F	72	H	CC	Yes	Yes	Yes	Yes	Yes	Yes										
166	F	84	H	CT	Yes	Yes	Yes	Yes	Yes	Yes										
167	F	79	H	CC	Yes	Yes	Yes	Yes	Yes	Yes										
168	F	94	H	CC	Yes	Yes	Yes	Yes	Yes	Yes										
169	F	84	H	CC	Yes	Yes	Yes	Yes	Yes	Yes										
170	F	84	H	CC	Yes	Yes	Yes	Yes	Yes	Yes										
171	F	68	H	CC	Yes	Yes	Yes	Yes		Yes										
172	F	80	H	CC	Yes	Yes	Yes	Yes		Yes										
173	F	86	H	CC	Yes	Yes	Yes	Yes	Yes	Yes										
174	F	89	H	CC	Yes	Yes	Yes	Yes	Yes	Yes										
175	F	91	H	CC	Yes	Yes	Yes	Yes	Yes	Yes										
176	F	82	H	CT	Yes	Yes	Yes	Yes		Yes										
177	F	80	H	CC	Yes	Yes	Yes	Yes		Yes										
178	F	83	H	CC	Yes	Yes	Yes	Yes	Yes	Yes										
179	F	84	H	CC	Yes	Yes	Yes	Yes	Yes	Yes										
180	F	82	H	CC	Yes	Yes	Yes	Yes		Yes										
181	M	85	H	CC													Yes			Yes
182	F	80	H	CC																Yes
183	F	95	H	CC																Yes
184	M	75	H	CC													Yes		Yes	Yes
185	M	79	H	CC																Yes
186	F	81	H	CT													Yes	Yes		
187	F	77	H	CC																Yes
188	M	86	H	CC													Yes		Yes	
189	F	62	H	CC															Yes	Yes
190	F	92	H	CC													Yes			
