# Supplementary material for: Functional characterisation of the osteoarthritis susceptibility locus at chromosome 6q14.1 marked by the polymorphism rs9350591
Source: BMC Med Genet. 2015 Sep 7;16:81. doi: 10.1186/s12881-015-0215-9 (PMC4562116; doi:10.1186/s12881-015-0215-9)
Supplement: Additional file 3: — n numbers used for real-time reverse transcription PCR (qPCR) and allelic expression imbalance (AEI). [file 12881_2015_215_MOESM3_ESM.pdf]

Additional file 3. *n* numbers used for real-time reverse transcription PCR (qPCR) and allelic expression imbalance (AEI)

		**Discovery qPCR (*n* numbers)**					
**Joint**	**rs9350591 genotype**	***COL12A1***	***TMEM30A***	***MYO6***	***SENP6***	***FILIP1***	***COX7A2***
OA hip	CC	14	14	14	14	12	14
	T carriers	7	7	7	7	7	7
OA knee	CC	36	36	36	36	33	36
	T carriers	17	17	17	17	16	17
NOF	CC	16	16	16	16	12	16
	T carriers	3	3	3	3	2	3

		**Replication qPCR (*n* numbers)**					
**Joint**	**rs9350591 genotype**	***COL12A1***	***TMEM30A***	***MYO6***	***SENP6***	***FILIP1***	***COX7A2***
OA hip	CC	15	18	13	16	7	19
	T carriers	6	6	6	6	4	6

		**AEI (*n* numbers)**			
**Joint**	**rs9350591 genotype**	***COL12A1***	***TMEM30A***	***MYO6***	***SENP6***
OA hip	CC	9	3	6	11
	T carriers	2	4	4	1
OA knee	CC	32	3	16	9
	T carriers	4	6	9	11
NOF	CC	4	1	4	11
	T carriers	1	1	0	0
