# Supplementary material for: Functional characterisation of the osteoarthritis susceptibility locus at chromosome 6q14.1 marked by the polymorphism rs9350591
Source: BMC Med Genet. 2015 Sep 7;16:81. doi: 10.1186/s12881-015-0215-9 (PMC4562116; doi:10.1186/s12881-015-0215-9)
Supplement: Additional file 4: — The seven transcript SNPs and their pair-wise D’ and r 2 values relative to rs9350591. [file 12881_2015_215_MOESM4_ESM.pdf]

Additional file 4. The seven transcript SNPs and their pair-wise D' and r^2 values relative to rs9350591

**Gene**	**Transcript SNP**	**SNP location**	**Alleles (major/minor)**	**MAF**	**Pairwise linkage disequilibrium relative to rs9350591**	
					r^2	D'
COL12A1	rs594012	Exon	T/A	0.092	0.018	1.000
COL12A1	rs240736	Exon	A/G	0.275	0.000	0.020
TMEM30A	rs41269315	3′ UTR	G/A	0.067	0.110	0.522
MYO6	rs1045758	3′ UTR	A/G	0.175	0.426	0.715
MYO6	rs699186	3′ UTR	C/T	0.357	0.079	1.000
SENP6	rs71561434	5′ UTR	C/T	0.133	0.746	0.925
SENP6	rs17414687	Exon	A/G	0.342	0.085	1.000
