# Supplementary material for: Functional characterisation of the osteoarthritis susceptibility locus at chromosome 6q14.1 marked by the polymorphism rs9350591
Source: BMC Med Genet. 2015 Sep 7;16:81. doi: 10.1186/s12881-015-0215-9 (PMC4562116; doi:10.1186/s12881-015-0215-9)
Supplement: Additional file 5: — Primer sequences used for genotyping and allelic expression imbalance by pyrosequencing (top table), and primer sequences and restriction enzyme used for genotyping by restriction fragment length polymorphism analysis (bottom table). [file 12881_2015_215_MOESM5_ESM.pdf]

Additional file 5. Primer sequences used for genotyping and allelic expression imbalance by pyrosequencing (top table), and primer sequences and restriction enzyme used for genotyping by restriction fragment length polymorphism analysis (bottom table)

SNP	Forward primer (5′ - 3′)	Reverse primer (5′ - 3′)	Sequencing primer (5′ - 3′)
rs594012	ACGCGATATTGCAGCACA	[Btn]ACACCTAACAGCCTCGATGTTC	CAGCACAGGTCCTGG
rs240736	[Btn]CTTGTGTTGTTAAGATAGGCA	TGAACCCCAACACCATCTAT	AGTCAGAAAGTGATGACCTG
rs41269315	[Btn]CAGGAGAGGTAGCAGGCTGAAC	ACTAGTGAGGAGGAGCAGCTGAG	GAGAGATAGGGTCAGTGAA
rs1045758	CCGCTGTAATTCCCAAAACTC	[Btn]ACTGCAAAGGAGGAAAAAAATAGA	CACTAATGTTTTGGTCTGAA
rs699186	[Btn]GGCATATTCTGATGTTTCTCATCC	AGTGCCTTGATCATTTTAAGTGGT	GTACTGTGCCATCCTTAA
rs71561434	CAAAGGCCTCCGCTGATG	[Btn]GCCCCTAGCTCGTTCTGCA	CACGCCTGGGCGGGGT
rs17414687	[Btn]CACCATCCACTGGAAAAGTAGAA	TTCTGGAATGCTTCGTAGTTCA	TCTGCAAGTATTTTCATTTA

SNP	Forward primer (5′ - 3′)	Reverse primer (5′ - 3′)	Restriction enzyme
rs9350591	CATAAGAAAGGCATGTTGC	CAGCTTTCATTGTATAACAAC	*Msp* I
