# Supplementary material for: Functional characterisation of the osteoarthritis susceptibility locus at chromosome 6q14.1 marked by the polymorphism rs9350591
Source: BMC Med Genet. 2015 Sep 7;16:81. doi: 10.1186/s12881-015-0215-9 (PMC4562116; doi:10.1186/s12881-015-0215-9)
Supplement: Additional file 6: — Table of OA and non-OA (Lonza) MSC donor characteristics used in chondrogenesis. [file 12881_2015_215_MOESM6_ESM.pdf]

Additional file 6. Table of OA and non-OA (Lonza) MSC donor characteristics used in chondrogenesis

Patient	Sex	Age	Joint	Disease
1	F	51	H	OA
2	F	61	H	OA
3	M	55	H	OA
4	F	24	H	Non-OA
5	F	41	H	Non-OA
6	M	25	H	Non-OA
