# Supplementary material for: Functional characterisation of the osteoarthritis susceptibility locus at chromosome 6q14.1 marked by the polymorphism rs9350591
Source: BMC Med Genet. 2015 Sep 7;16:81. doi: 10.1186/s12881-015-0215-9 (PMC4562116; doi:10.1186/s12881-015-0215-9)
Supplement: Additional file 7: — Bioinformatics database search of the SNPs in high LD with rs9350591. [file 12881_2015_215_MOESM7_ESM.pdf]

Additional file 7. Bioinformatics database search of the SNPs in high LD with rs9350591

								Chromatin State		
**SNP**	**Distance from rs9350591 (bp)**	**r^2 relative to rs9350591**	**D' relative to rs9350591**	**Genotyped on arcOGEN array**	**HG19 co-ordinates**	**SNP location**	**Transcription factor binding**	**Bone marrow-derived cultured MSCs**	**MSC-derived chondrocyte cultured cells**	**Osteoblast primary cells**
rs12211255	53197	0.810	1.000	No	76188330	Intronic	No data	No data	No data	No data
rs10943249	42308	1.000	1.000	No	76199219	Intronic	No data	No data	No data	No data
rs35985089	39845	1.000	1.000	No	76201682	Intronic	No data	Weak transcription	Weak repressed polycomb	Weak repressed polycomb
rs9343292	38871	1.000	1.000	No	76202656	Intronic	ATF2, NFKB1, POLR2A, TBP, NR3C1, USF1	Weak transcription	Flanking active TSS	Repressed polycomb
rs11964634	21727	1.000	1.000	No	76219800	Intergenic	No data	No data	No data	No data
rs9360913	17208	0.935	1.000	No	76224319	Intergenic	No data	Quiescent/low	Quiescent/low	Quiescent/low
rs9359125	10223	0.872	1.000	No	76231304	Intergenic	No data	Quiescent/low	Quiescent/low	Quiescent/low
rs12190734	9543	0.935	1.000	No	76231984	Intergenic	No data	No data	No data	No data
rs9343297	6830	0.935	1.000	No	76234697	Intergenic	EP300, TCF7L2, TRIM28, ZNF263, GATA3, REST	Enhancer	Enhancer	Enhancer
rs9341526	5138	0.935	1.000	No	76236389	Intergenic	ARID3A, CTCF, CEBPB, FOXM1, JUND, SMC3, YY1, RAD21, RUNX3	Quiescent/low	Quiescent/low	Weak repressed polycomb
rs12200169	4406	1.000	1.000	No	76237121	Intergenic	No data	Quiescent/low	Quiescent/low	Weak repressed polycomb
rs12207675	3786	0.935	1.000	No	76237741	Intergenic	No data	Quiescent/low	Quiescent/low	Weak repressed polycomb
rs12202443	2913	0.935	1.000	No	76238614	Intergenic	CTCF	Quiescent/low	Quiescent/low	Weak repressed polycomb
rs13192994	2265	0.935	1.000	No	76239262	Intergenic	No data	No data	No data	No data
rs9343299	1682	0.935	1.000	No	76239845	Intergenic	No data	No data	No data	No data
rs9352215	1646	0.935	1.000	No	76239881	Intergenic	No data	No data	No data	No data
rs9350591	0	1.000	1.000	Yes	76241527	Intergenic	No data	Weak repressed polycomb	Quiescent/low	Weak repressed polycomb
rs117337795	3443	0.935	1.000	No	76244970	Intergenic	No data	No data	No data	No data
rs7756065	5985	1.000	1.000	Yes	76247512	Intergenic	No data	Weak repressed polycomb	Weak repressed polycomb	Weak repressed polycomb
rs9359127	6118	1.000	1.000	No	76247645	Intergenic	No data	Weak repressed polycomb	Repressed polycomb	Weak repressed polycomb
rs9359128	8929	1.000	1.000	No	76250456	Intergenic	No data	Weak repressed polycomb	Weak repressed polycomb	Weak repressed polycomb
rs11963619	11538	1.000	1.000	No	76253065	Intergenic	No data	No data	No data	No data
rs9352217	17598	0.810	1.000	No	76259125	Intergenic	No data	Quiescent/low	Quiescent/low	Weak repressed polycomb
rs9360921	24115	0.810	1.000	No	76265642	Intergenic	USF1	Quiescent/low	Weak repressed polycomb	Weak repressed polycomb
rs12201305	42095	0.810	1.000	No	76283622	Intergenic	No data	No data	No data	No data
rs12213476	49846	0.810	1.000	No	76291373	Intergenic	No data	Quiescent/low	Quiescent/low	Quiescent/low
rs9360926	58467	0.810	1.000	No	76299994	Intergenic	No data	No data	No data	No data
rs9359133	84148	0.810	1.000	No	76325675	Intronic	No data	Quiescent/low	Quiescent/low	Quiescent/low
rs67016585	88824	0.810	1.000	No	76330351	Intronic	GATA1	Flanking active TSS	Enhancer	Enhancer
rs33997653	91407	0.810	1.000	No	76332934	Intronic	No data	No data	No data	No data
rs12214738	102256	0.810	1.000	No	76343783	Intronic	No data	Strong transcription	Strong transcription	Strong transcription
rs9360930	139150	0.810	1.000	No	76380677	Intronic	No data	Strong transcription	Strong transcription	Strong transcription
rs9350596	139519	0.810	1.000	No	76381046	Intronic	No data	No data	No data	No data
rs9341531	140538	0.810	1.000	No	76382065	Intronic	No data	Strong transcription	Strong transcription	Weak transcription
rs9360932	146410	0.810	1.000	No	76387937	Intronic	No data	No data	No data	No data
rs12192223	156682	0.810	1.000	No	76398209	Intronic	No data	No data	No data	No data
rs9343320	174156	0.810	1.000	No	76415683	Intronic	No data	Weak transcription	Strong transcription	Weak transcription
rs12208368	176259	0.810	1.000	No	76417786	Intronic	No data	Strong transcription	Strong transcription	Weak transcription
rs12212171	178209	0.810	1.000	No	76419736	Intronic	No data	Strong transcription	Enhancer	Weak transcription
rs17792773	186968	0.810	1.000	No	76428495	Intergenic	No data	Strong transcription	Strong transcription	Quiescent/low
