# Supplementary material for: Functional characterisation of the osteoarthritis susceptibility locus at chromosome 6q14.1 marked by the polymorphism rs9350591
Source: BMC Med Genet. 2015 Sep 7;16:81. doi: 10.1186/s12881-015-0215-9 (PMC4562116; doi:10.1186/s12881-015-0215-9)
Supplement: Additional file 8: — OA vs NOF analysis of qPCR data for hip and knee cartilage combined. (PDF 75 kb) [file 12881_2015_215_MOESM8_ESM.pdf]

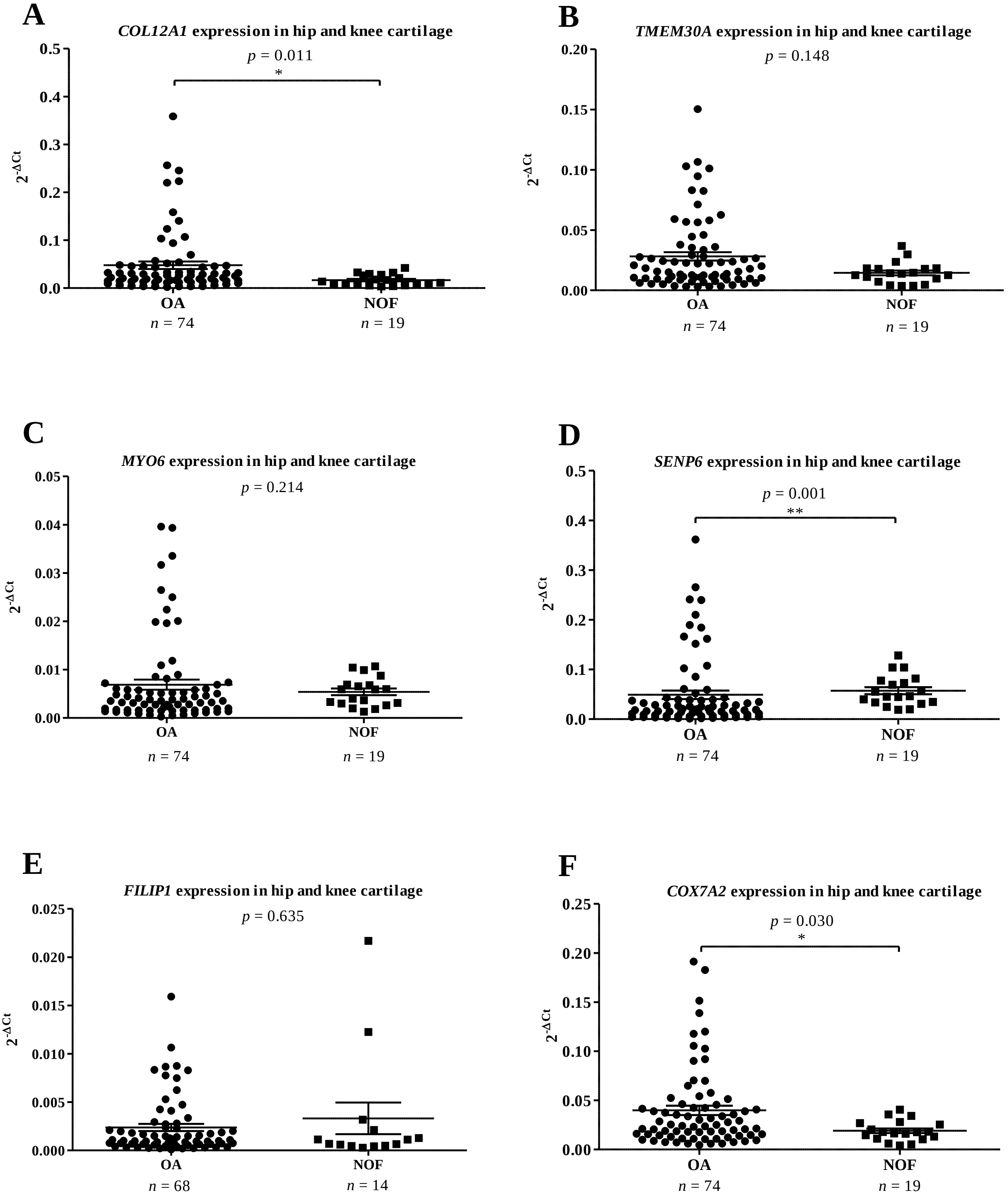

Additional file 8. OA vs NOF analysis of qPCR data for hip and knee cartilage combined. The qPCR data for hip and knee overall gene expression were combined and compared to gene expression levels in the NOF cohort for (A) *COL12A1*, (B) *TMEM30A*, (C) *MYO6*, (D) *SENP6*, (E) *FILIP1* and (F) *COX7A2*. The expression of *COL12A1* and *COX7A2* were both significantly increased in OA hip and knee relative to NOF. The expression of *SENP6* was significantly decreased in OA hip and knee relative to NOF. The horizontal lines represent the mean and the error bars represent the SEM. Statistical significance was assessed using the Mann-Whitney *U* test and is not corrected for multiple testing. * $p < 0.05$; ** $p < 0.01$.
