# Supplementary material for: Functional characterisation of the osteoarthritis susceptibility locus at chromosome 6q14.1 marked by the polymorphism rs9350591
Source: BMC Med Genet. 2015 Sep 7;16:81. doi: 10.1186/s12881-015-0215-9 (PMC4562116; doi:10.1186/s12881-015-0215-9)
Supplement: Additional file 9: — Genotype analysis of qPCR data for hip and knee cartilage combined. (PDF 64 kb) [file 12881_2015_215_MOESM9_ESM.pdf]

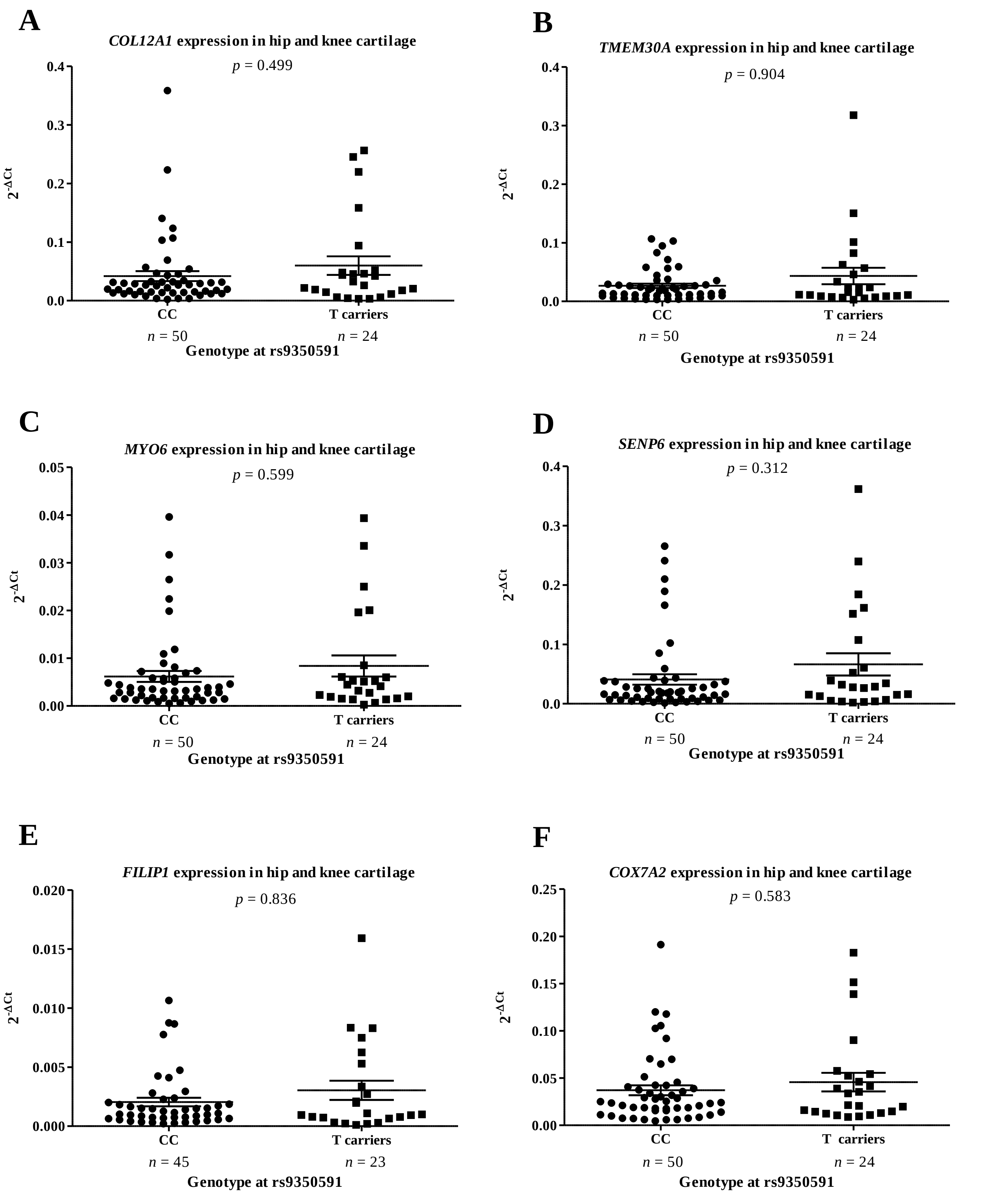

Additional file 9. Genotype analysis of qPCR data for hip and knee cartilage combined. The qPCR data for OA hip and OA knee overall gene expression were combined and stratified by rs9350591 genotype for (A) *COL12A1*, (B) *TMEM30A*, (C) *MYO6*, (D) *SENP6*, (E) *FILIP1* and (F) *COX7A2*.We observed no significant differences in gene expression relative to genotype at rs9350591. The horizontal lines represent the mean and the error bars represent the SEM. Statistical significance was assessed using the Mann-Whitney *U* test and is not corrected for multiple testing.
