# Supplementary material for: Functional characterisation of the osteoarthritis susceptibility locus at chromosome 6q14.1 marked by the polymorphism rs9350591
Source: BMC Med Genet. 2015 Sep 7;16:81. doi: 10.1186/s12881-015-0215-9 (PMC4562116; doi:10.1186/s12881-015-0215-9)
Supplement: Additional file 10: — Diagram to explain the premise of allelic expression imbalance analysis when there are no transcript SNPs in high LD ( r 2 ≥ 0.8) with the association SNP. (PDF 89 kb) [file 12881_2015_215_MOESM10_ESM.pdf]

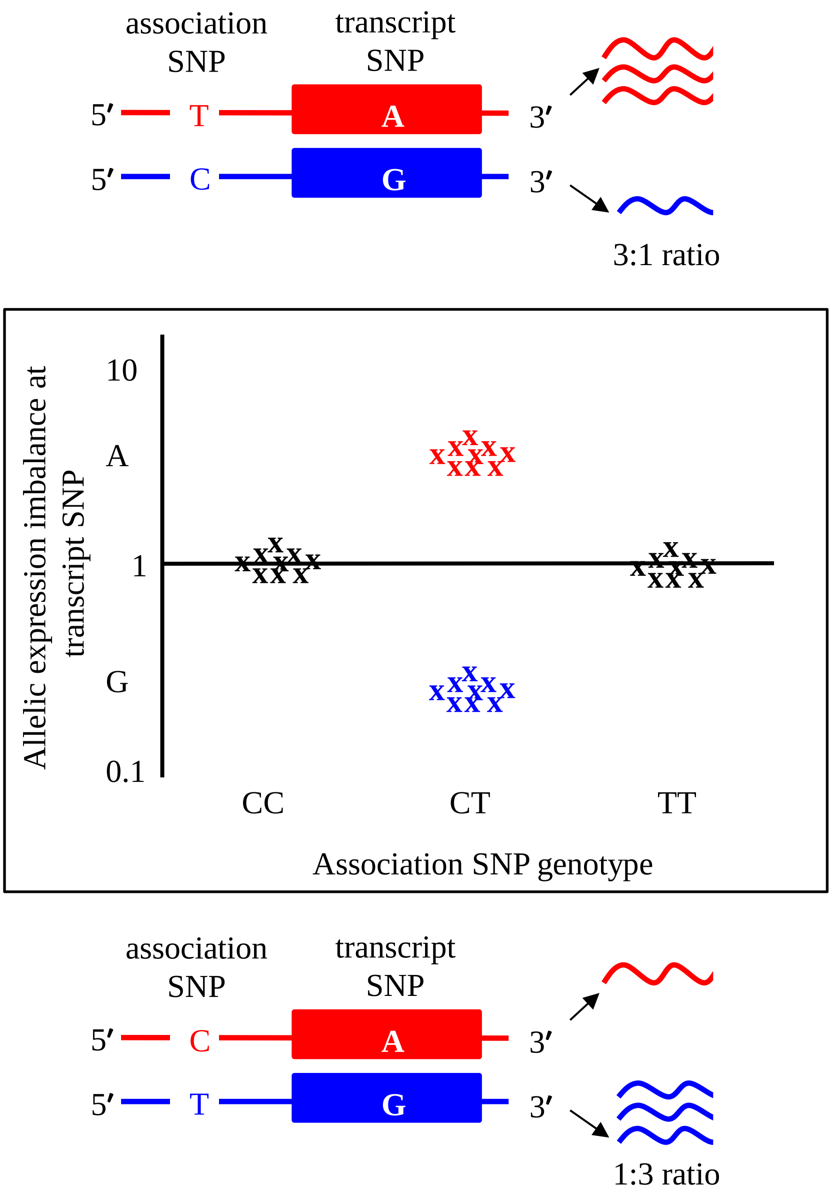

Additional file 10. Diagram to explain the premise of allelic expression imbalance analysis when there are no transcript SNPs in high LD ($r^2 \geq 0.8$) with the association SNP. For compound heterozygotes, two different haplotypes can occur: the minor (risk) allele of the association SNP with the minor allele of the transcript SNP, or the minor (risk) allele of the association SNP with the major allele of the transcript SNP. Regardless of the haplotype, if the risk allele is driving an imbalance, it would be reflected in the allelic output of the transcript SNP. This would cause an imbalance of the allelic output, which would transpire as clustering above and below $y = 1$. Homozygotes at the association SNP would cluster around $y = 1$, as there would be no allelic differences at the SNP to drive a transcript imbalance.
