# Supplementary material for: Functional characterisation of the osteoarthritis susceptibility locus at chromosome 6q14.1 marked by the polymorphism rs9350591
Source: BMC Med Genet. 2015 Sep 7;16:81. doi: 10.1186/s12881-015-0215-9 (PMC4562116; doi:10.1186/s12881-015-0215-9)
Supplement: Additional file 11: — Allelic expression imbalance analysis stratified by disease state and joint site. (PDF 57 kb) [file 12881_2015_215_MOESM11_ESM.pdf]

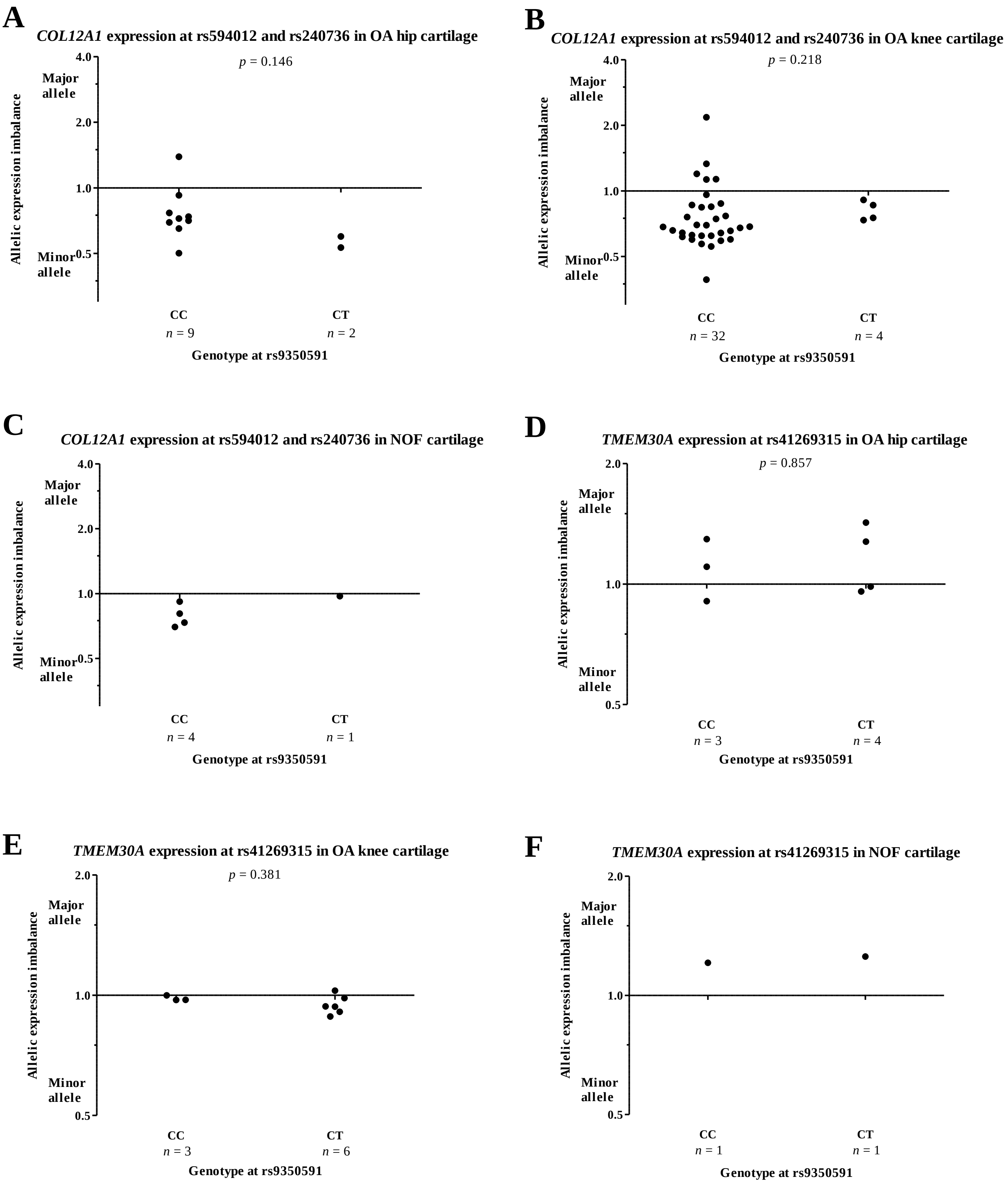
A
COL12A1 expression at rs594012 and rs240736 in OA hip cartilage
p = 0.146
Allelic expression imbalance
4.0
2.0
1.0
0.5
Major allele
Minor allele
CC
n = 9
CT
n = 2
Genotype at rs9350591
B
COL12A1 expression at rs594012 and rs240736 in OA knee cartilage
p = 0.218
Allelic expression imbalance
4.0
2.0
1.0
0.5
Major allele
Minor allele
CC
n = 32
CT
n = 4
Genotype at rs9350591
C
COL12A1 expression at rs594012 and rs240736 in NOF cartilage
Allelic expression imbalance
4.0
2.0
1.0
0.5
Major allele
Minor allele
CC
n = 4
CT
n = 1
Genotype at rs9350591
D
TMEM30A expression at rs41269315 in OA hip cartilage
p = 0.857
Allelic expression imbalance
2.0
1.0
0.5
Major allele
Minor allele
CC
n = 3
CT
n = 4
Genotype at rs9350591
E
TMEM30A expression at rs41269315 in OA knee cartilage
p = 0.381
Allelic expression imbalance
2.0
1.0
0.5
Major allele
Minor allele
CC
n = 3
CT
n = 6
Genotype at rs9350591
F
TMEM30A expression at rs41269315 in NOF cartilage
Allelic expression imbalance
2.0
1.0
0.5
Major allele
Minor allele
CC
n = 1
CT
n = 1
Genotype at rs9350591

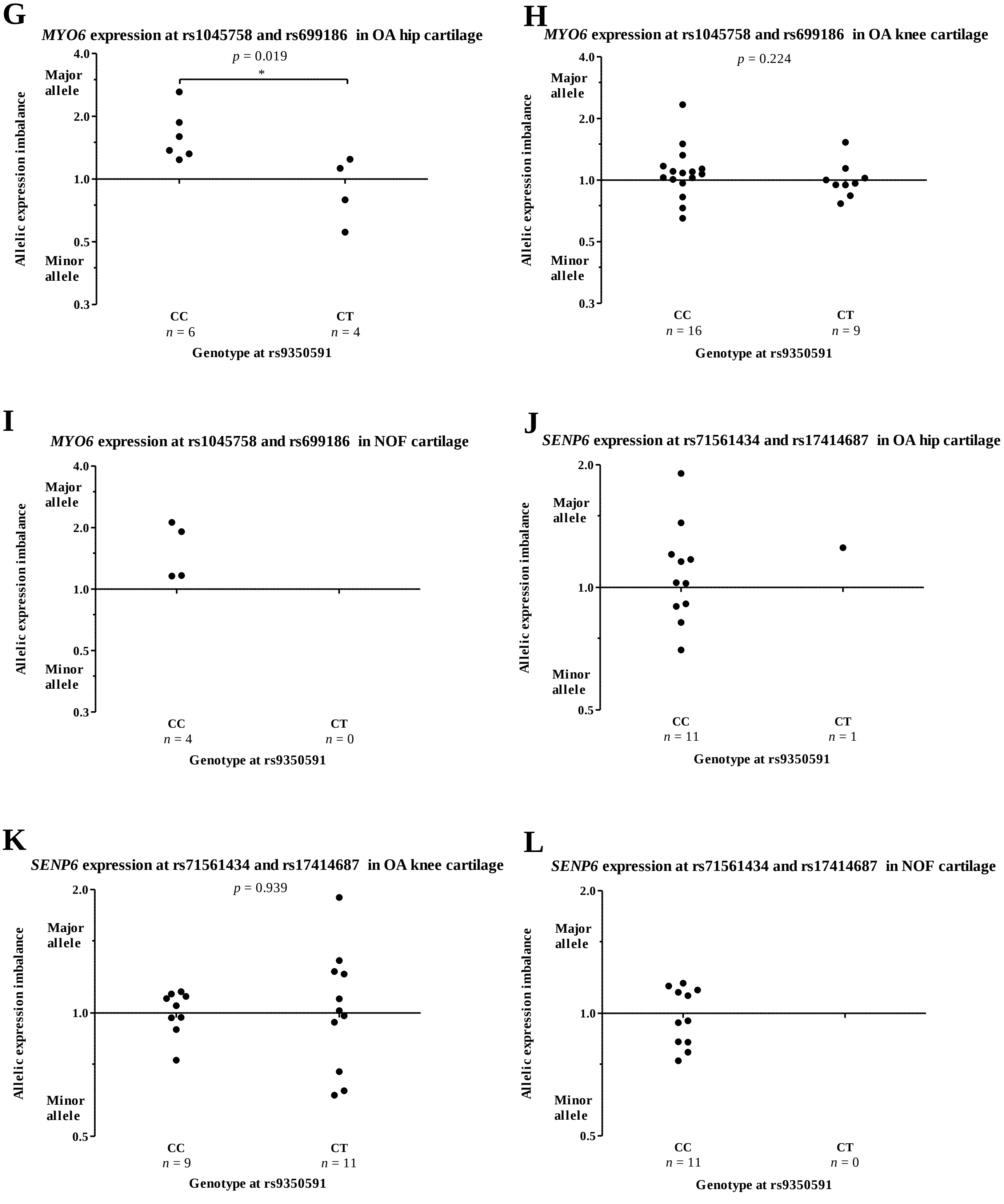

Additional file 11. Allelic expression imbalance analysis stratified by disease state and joint site. The data were stratified by OA hip, OA knee and NOF for *COL12A1* (A, B and C), *TMEM30A* (D, E and F), *MYO6* (G, H and I), and *SENP6* (J, K and L). Stratification did not result in any changes to the analysis of combined data. Statistical significance was assessed using the Mann-Whitney *U* test and is not corrected for multiple testing.
